# Supplementary material for: Content-rich biological network constructed by mining PubMed abstracts
Source: BMC Bioinformatics. 2004 Oct 8;5:147. doi: 10.1186/1471-2105-5-147 (PMC528731; doi:10.1186/1471-2105-5-147)
Supplement: Additional File 2 — The original results of the above study (non-essential files are deleted to keep the file size under the limit set by BMC bioinformatics). [file 1471-2105-5-147-S2.bz2 › chilibotAdditionalFile2/dip30/98ID7768895E416/html/SNAP25_STX4A.html]

 


 **SNAP25** and **STX4A** 
  
Found 24 abstracts in PubMed, retrieved 24.  
 

 What does Google say? 
 PDF only 
| .edu only 

---

**Interactive relationship** (e.g. stimulation, inhibition, etc)

**Neutral relationship**- The biochemical consequences of SNARE protein phosphorylation included a reduced interaction between SNAP 25  [ **SNAP25** ]  and phosphorylated syntaxin 4  [ **STX4A** ]  and an enhanced interaction between phosphorylated syntaxin 1A and the synaptic vesicle protein synaptotagmin I, a potential Ca2 sensor in triggering synaptic vesicle exocytosis.  Ref: 9930733 J Neurochem, 1999
- This platelet Sec1 protein PSP bound to syntaxin 4  [ **STX4A** ]  and thereby excluded the binding of SNAP 25  [ **SNAP25** ]  with syntaxin 4  [ **STX4A** ] , an interaction critical to vesicle docking.  Ref: 10194441 Blood, 1999
- Synaptosome associated 23 kDa protein SNAP23 is a widely expressed isoform of  **SNAP25** , the principal t SNARE of neuronal cells, and colocalizes with syntaxin4  [ **STX4A** ]  in the plasma membrane of 3T3 L1 adipocytes.  Ref: 10713150 J Biol Chem, 2000

**Non-interactive relationship** (e.g. studied together, co-existance, homology, etc.)

- Syntaxin 4  [ **STX4A** ]  and SNAP 25  [ **SNAP25** ]  were also detected.  Ref: 11162495 Biochem Biophys Res Commun, 2001
- A combination of reverse transcription polymerase chain reaction and northern and western blotting is used to show expression of the target membrane SNARE, proteins SNAP 25  [ **SNAP25** ]  and syntaxin 4  [ **STX4A** ]  and the vesicular SNARE protein VAMP, the minimum molecular requirements for core exocytotic complex formation.  Ref: 11472886 Bone, 2001
- The plasma membranes of insulin responsive cells have also been shown to contain syntaxin 4  [ **STX4A** ]  and the 25 kDa synaptosome associated protein SNAP 25  [ **SNAP25** ] , two proteins that form a complex with VAMP 2.  Ref: 9164859 Biochem J, 1997
- whereas SNAP 25  [ **SNAP25** ]  on its own bound poorly to these vesicles, the syntaxin 4  [ **STX4A** ]  SNAP 25  [ **SNAP25** ]  complex gave a strong interaction.  Ref: 8760387 Biochem J, 1996
- The potential functional involvement of VAMP 2, SNAP 25  [ **SNAP25** ]  and syntaxin 4  [ **STX4A** ]  in the trafficking of GLUT4 was assessed in the present study by determining the effect on GLUT4 translocation of microinjection of toxins that specifically cleave VAMPs or SNAP 25  [ **SNAP25** ] , or microinjection of specific peptides from VAMP 2 and syntaxin 4  [ **STX4A** ] .  Ref: 9164859 Biochem J, 1997
- However, when vesicle associated membrane protein VAMP 2 was immunoprecipitated from lysates of parotid acinar cells, syntaxin 4  [ **STX4A** ]  and SNAP 23 were not coprecipitated with VAMP 2, although syntaxin 1 and SNAP 25  [ **SNAP25** ] , t SNAREs in neuronal cells, were clearly coprecipitated with VAMP 2 from brain lysates.  Ref: 10739858 Arch Oral Biol, 2000
- We found that platelet membranes contain a full complement of interacting proteins VAMP, SNAP 25  [ **SNAP25** ] , and syntaxin 4  [ **STX4A** ]  that are necessary for vesicle docking and fusion with the plasma membrane.  Ref: 10194441 Blood, 1999
- It describes the isolation and characterization of cDNA molecules encoding SNAP 25  [ **SNAP25** ]  A and B isoforms, syntaxin 4  [ **STX4A** ]  and synaptobrevins from two major human insulin responsive tissues, skeletal muscle and fat.  Ref: 8760387 Biochem J, 1996
- Syntaxin 4  [ **STX4A** ]  and SNAP 25  [ **SNAP25** ]  were also detected.  Ref: 11263991 Biochem Biophys Res Commun, 2001
- Syntaxin 4  [ **STX4A** ] , SNAP 23 and or SNAP 25  [ **SNAP25** ]  are also implicated in this process.  Ref: 9268721 Biochem Biophys Res Commun, 1997
